# Antiviral Drugs Screening for Swine Acute Diarrhea Syndrome Coronavirus

**DOI:** 10.3390/ijms231911250

**Published:** 2022-09-24

**Authors:** Yangzhen Chen, Yecheng You, Shuqi Wang, Lin Jiang, Lili Tian, Shaozhou Zhu, Xiaoping An, Lihua Song, Yigang Tong, Huahao Fan

**Affiliations:** College of Life Science and Technology, Beijing University of Chemical Technology, Beijing 100029, China

**Keywords:** antiviral drugs, coronavirus, SADS-CoV, drug screening

## Abstract

Coronaviruses as possible cross-species viruses have caused several epidemics. The ongoing emergency of coronavirus disease 2019 (COVID-19) caused by SARS-CoV-2 has posed severe threats to the global economy and public health, which has generated great concerns about zoonotic viruses. Swine acute diarrhea syndrome coronavirus (SADS-CoV), an alpha-coronavirus, was responsible for mass piglet deaths, resulting in unprecedented economic losses, and no approved drugs or vaccines are currently available for SADS-CoV infection. Given its potential ability to cause cross-species infection, it is essential to develop specific antiviral drugs and vaccines against SADS-CoV. Drug screening was performed on a total of 3523 compound-containing drug libraries as a strategy of existing medications repurposing. We identified five compounds (gemcitabine, mycophenolate mofetil, mycophenolic acid, methylene blue and cepharanthine) exhibiting inhibitory effects against SADS-CoV in a dose-dependent manner. Cepharanthine and methylene blue were confirmed to block viral entry, and gemcitabine, mycophenolate mofetil, mycophenolic acid and methylene blue could inhibit viral replication after SADS-CoV entry. This is the first report on SADS-CoV drug screening, and we found five compounds from drug libraries to be potential anti-SADS-CoV drugs, supporting the development of antiviral drugs for a possible outbreak of SADS-CoV in the future.

## 1. Introduction

Since the outbreak of coronavirus disease 2019 (COVID-19) in late December 2019, coronaviruses have returned to the limelight. In the ongoing COVID-19 pandemic, the consistent emergence of deadly coronavirus diseases and different variants have raised concerns about the transmission and evolution of coronavirus, especially the cross-species transmission, resulting in zoonotic diseases which may pose threats to human health in the future [1].

Swine acute diarrhea syndrome coronavirus (SADS-CoV), belonging to the genus *Alphacoronavirus*, was first isolated in 2017 in Guangdong province, China, and re-emerged in 2019, responsible for the outbreaks of severe porcine diarrhea in local farms, causing mass piglet deaths and economic losses [2,3]. SADS-CoV shares 95% genome identity to bat coronavirus HKU2 discovered in 2004, and SADS-related coronaviruses have previously spread in *Rhinolophus affinis* and *Rhinolophus sinicus* [4]. Based on phylogenetic analysis, the spike (S) glycoproteins of SADS-CoV and related coronaviruses are distinct from other α-coronaviruses but are more closely related to β-coronaviruses [5,6]. As an important role in mediating coronavirus entry, the unique structure of SADS-CoV S glycoprotein was speculated to be the result of α- and β-coronavirus S gene recombination, suggesting an evolutionary recombination in coronavirus.

SADS-CoV can infect various kinds of cell lines originating from different species including, humans, bats, pigs, monkeys, chickens, mice, and nonhuman primates [7,8]. Notably, SADS-CoV also infects a variety of human immortalized cell lines, including primary human lung and intestinal cells, implying the potential susceptibility of humankind to the SADS-CoV virus [9]. Besides, none of the known host receptors like aminopeptidase N (APN), dipeptidyl-peptidase 4 (DPP4), and angiotensin-converting enzyme 2 (ACE2) is used for SADS-CoV entry, indicating the existence of unknown receptors for SADS-CoV entry [4,8]. The unique evolutionary relationship and the possibility of cross-species transmission pose SADS-CoV as a potential threat to human beings. Unfortunately, the limited spread and its effects resulted in insufficient research and focus on SADS-CoV, and no approved vaccines or therapeutics is currently available for treatment.

Given the potential for cross-species transmission and threat to human health, it is important to establish a system to prevent the outbreak of SADS-CoV or related viruses, especially in the current context of the COVID-19 pandemic. To quickly find the antiviral drugs, we performed a drug screening of a total of 3523 molecules from an approved drug library, an antiviral compound library, and an anti-COVID-19 traditional Chinese medicine compound library. We discovered five compounds as potential anti-SADS-CoV drugs for treatments in the case of any outbreaks of SADS-CoV or related viruses in the future.

## 2. Results

### 2.1. Antiviral Drug Screening and Evaluation

**Figure 1:**
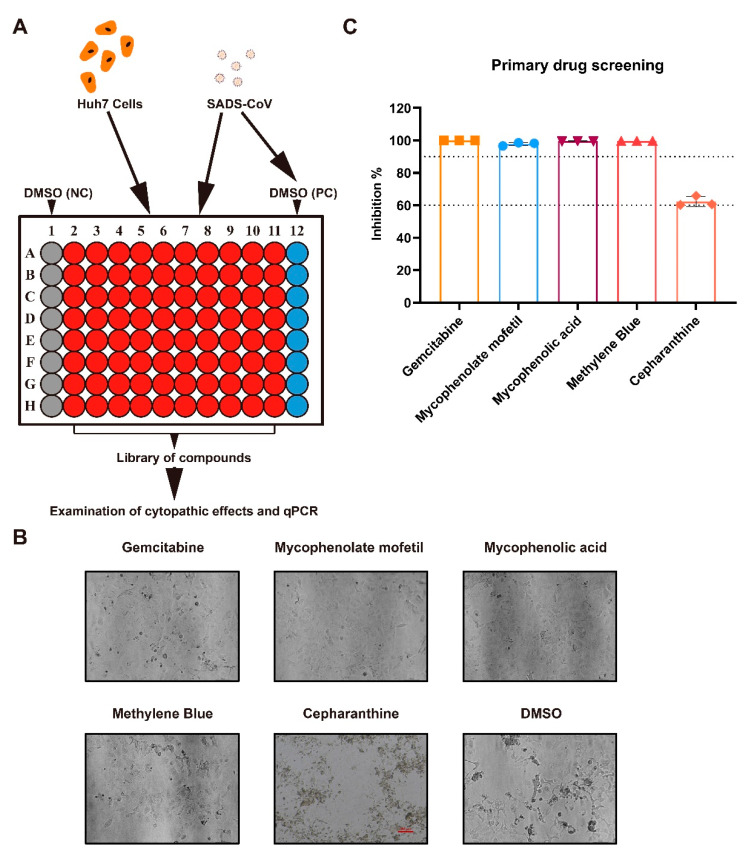
Primary screening for anti-SADS-CoV compounds. (**A**) Overview of antiviral compound screening. Huh7 cells were seeded in 96-well plates, then treated with SADS-CoV (MOI 0.1) and each compound to a final concentration of 10 μM (containing less than 0.1% DMSO). (**B**) The representative images of CPE on Huh7 cells incubated with SADS-CoV and the five compounds. Images were captured at 48 h post-infection (hpi) under 10× objective. (**C**) The inhibitory rates of gemcitabine, mycophenolate mofetil, mycophenolic acid, methylene blue and cepharanthine are shown. Cells incubated with dimethyl sulfoxide (DMSO) and virus were regarded as the positive control (PC), and negative control was treated with 0.1% DMSO only. The viral RNA expression levels in the cells were quantified by qPCR and normalized to GAPDH, and the compound inhibitory rates were calculated compared to PC. Data represent the mean of the three replicate results. Error bars represent ±1 SD, and the experiments were repeated at least twice.

To quickly find specific pharmacotherapy for SADS-CoV infection, we used Huh7 cells and approved drug libraries to screen antiviral drugs by measuring the viral-induced cytopathic effect (CPE) and the expression level of viral RNA in the cells (Figure 1A), which has been applied to screen antiviral drugs for other viruses [10,11]. Of the total 3523 compounds tested, 5 of them significantly exhibited CPE inhibitions in SADS-CoV infection at 10 μM, including gemcitabine, mycophenolate mofetil, mycophenolic acid, cepharanthine and methylene blue (Figure 1B). Meanwhile, the qPCR analysis showed that at 10 μM, these five drugs inhibited SADS-CoV viral replication in Huh7 cells, with an inhibition rate of more than 60% (Figure 1C). These preliminary results suggested that cepharanthine, gemcitabine, mycophenolate mofetil, mycophenolic acid, and methylene blue were potential antiviral drugs for SADS-CoV infection.

**Figure 2:**
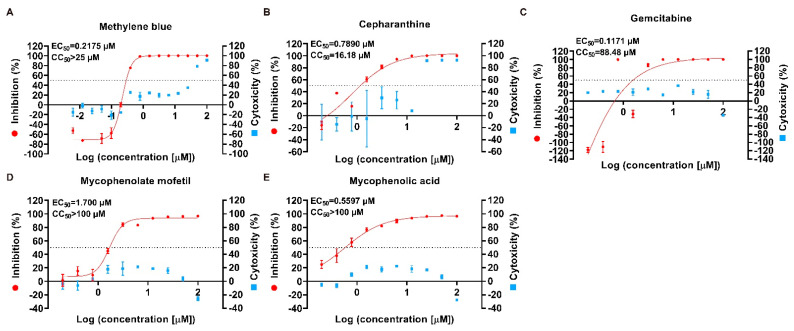
Validations of antiviral drugs selected from primary screening. Two-fold serial dilutions of methylene Blue, cepharanthine, gemcitabine, mycophenolate mofetil, and mycophenolic acid were separately added to the cells for antiviral activity and cytotoxicity test (**A**–**E**). The positive control was treated with the virus and 0.1% DMSO, while only 0.1% DMSO-containing media were added to the negative control. Cell viability and viral RNA expression levels were separately assayed by CellTiter-Blue and qPCR. Inhibitory rate and cytotoxicity were evaluated, compared with the positive and negative control, respectively. Dose-response curves were generated, and EC_50_ and CC_50_ values were calculated by using nonlinear regression analysis. Data represent the mean of the three replicate results. Error bars represent ±1 SD, and the experiments were repeated at least twice.

Furthermore, we evaluated the cytotoxicity and inhibitory effects of the five compounds to determine their antiviral activity. The inhibition rate of these five drugs on cell infection was reduced with decreasing concentrations in a dose-dependent manner (Figure 2). The 50% effective concentration (EC_50_) which represented the effective antiviral activity were all less than 2 μM, and even 0.1171 μM of gemcitabine was enough for inhibiting 50% of cell infection. (Figure 2). Although the 50% cytotoxic concentration (CC_50_) of cepharanthine was 16.18 μM showing some cytotoxicity, gemcitabine, mycophenolate mofetil, mycophenolic acid, and methylene blue exhibited low cytotoxicity. In general, the selectivity index (SI) of these five drugs was more than 10, suggesting their effectiveness and safety for SADS-CoV infection (Table 1).

### 2.2. Preliminary Identification of Potential Antiviral Targets

**Figure 3:**
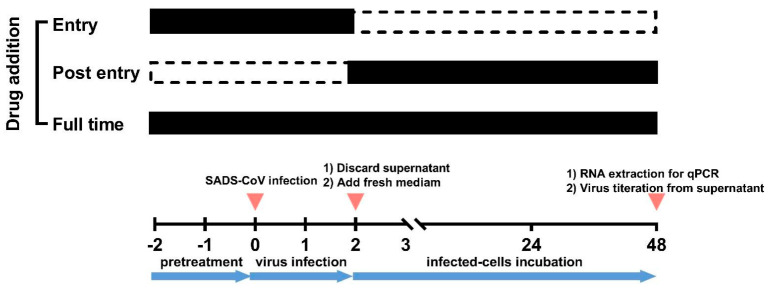
Procedure of the time-of-addition assay. Cells were divided into four groups with different treatments: (1) Before infection, cells of “Entry” and “Full time” groups were incubated with the indicated compounds for 2 h. (2) Cells were incubated with the indicated compounds and SADS-CoV (MOI 0.1) in the “Entry” and “Full time” groups, while cells in the “Post entry” and positive control groups were treated with the virus only. (3) After 2 h-infection, the media were replaced with fresh media with the indicated compounds in the “Post entry” and “Full time” groups, while other groups were supplemented with media without compounds. Media of the positive control groups always contained 0.1% DMSO. After 48 hpi, the supernatant and cellular RNA were collected for evaluation.

The lifecycle of coronavirus comprises viral entry (attachment, internalization, and fusion), gene expression, RNA synthesis, particle assembly, and release [12]. To determine the potential inhibition targets of drugs for SADS-CoV infection, we compared the efficacy of drug intervention at different stages of SADS-CoV infection. Methylene blue (1.5625 μM), mycophenolate mofetil (12.5 μM), mycophenolic acid (12.5 μM), gemcitabine (6.25 μM), and cepharanthine (6.25 μM) were separately added to the cells during the stage of virus “Entry”, “Post entry” and “Full time” (Figure 3).

**Figure 4:**
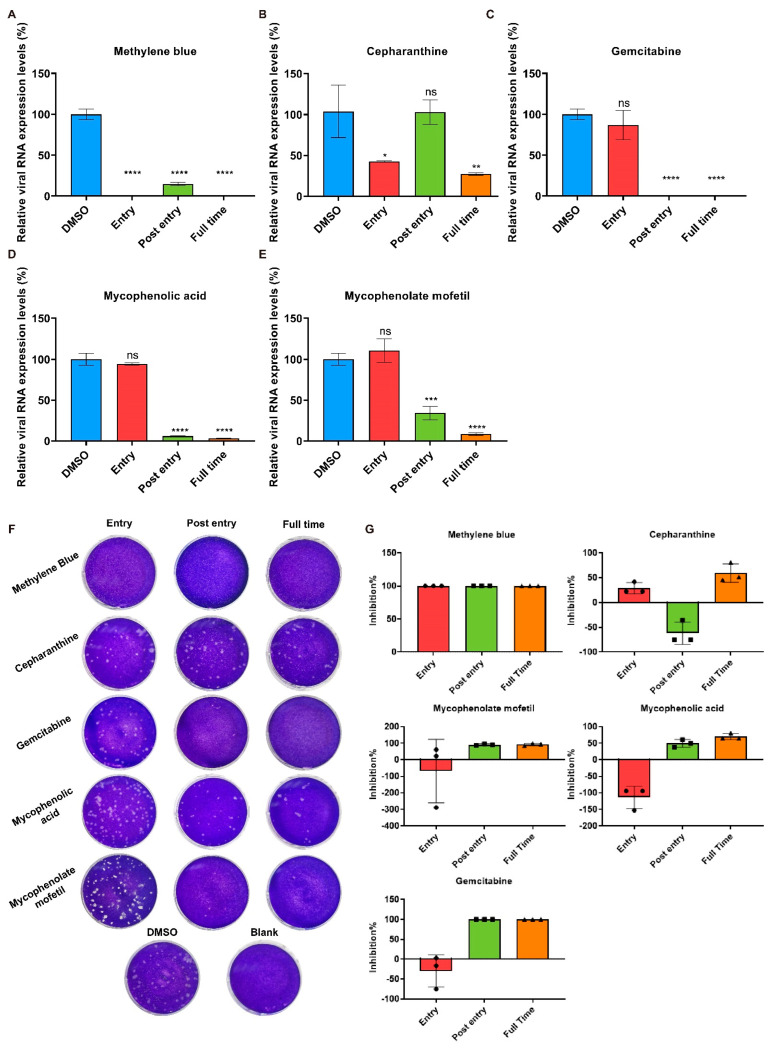
Preliminary identification of potential antiviral targets. Methylene blue (1.5625 μM), mycophenolate mofetil (12.5 μM), mycophenolic acid (12.5 μM), gemcitabine (6.25 μM), and cepharanthine (6.25 μM) were separately tested through the time-of-addition assay. qPCR and plaque assay were used to examine viral RNA expression levels in the cells (**A**–**E**) and infectious progeny in the supernatant (**F**), respectively. The viral titers in the supernatant were quantified and used to calculate the inhibition rates (**G**). DMSO was added as the positive control and the RNA expression levels were normalized to the positive control. The shown results are representative of one experiment out of at least two experiments. Error bars represent ±1 SD; ns, no significant difference; * *p* < 0.05, ** *p* < 0.01, *** *p* < 0.001, **** *p* < 0.0001 by Student’s *t*-test.

Apparently, the addition of these five compounds during the whole 48 h infection could effectively reduce cell cytopathy (Figure S1). As validated by the qPCR, the quantification of viral RNA expression levels in the cells showed that methylene blue and gemcitabine were both excellent inhibitors and could decrease more than 99.9% of the viral RNA expression in the cells at ”Full time” addition (Figure 4A,C). The addition of mycophenolate mofetil, mycophenolic acid, and cepharanthine at “Full time” reduced the viral RNA expression by 91.5%, 96.7% and 72.9%, respectively (Figure 4B,D,E). Additionally, the viral RNA expression levels in the “Entry” groups of methylene blue and cepharanthine were lower than that of the positive control, showing a 99.8% and 57.5% decrease, respectively. In the “Post entry” groups, methylene blue caused a reduction of approximately 85.4% in the viral RNA expression, while no significant difference was observed with cepharanthine addition (Figure 4A,B). Meanwhile, the addition of mycophenolate mofetil, mycophenolic acid, and gemcitabine could effectively decrease the viral RNA expression in the “Post entry” groups, by 65.8%, 93.9%, and 99.9%, respectively, but less remarkable differences were observed in the “Entry” groups (Figure 4C–E). The viral titers in the supernatant demonstrated similar results that the addition of cepharanthine at the ”Entry” stage could reduce infectious progeny, and mycophenolate mofetil, mycophenolic acid, and gemcitabine exhibited inhibitory effects only when they were added after viral entry; but methylene blue showed strong inhibitory activity against viral replication during both periods (Figure 4F,G).

These results indicated that cepharanthine might inhibit SADS-CoV entry in cells and methylene blue could inhibit both viral entry and viral process in cells, while mycophenolate mofetil, mycophenolic acid, and gemcitabine inhibited viral replication after SADS-CoV entry.

### 2.3. Cepharanthine Inhibits SADS-CoV Entry

**Figure 5:**
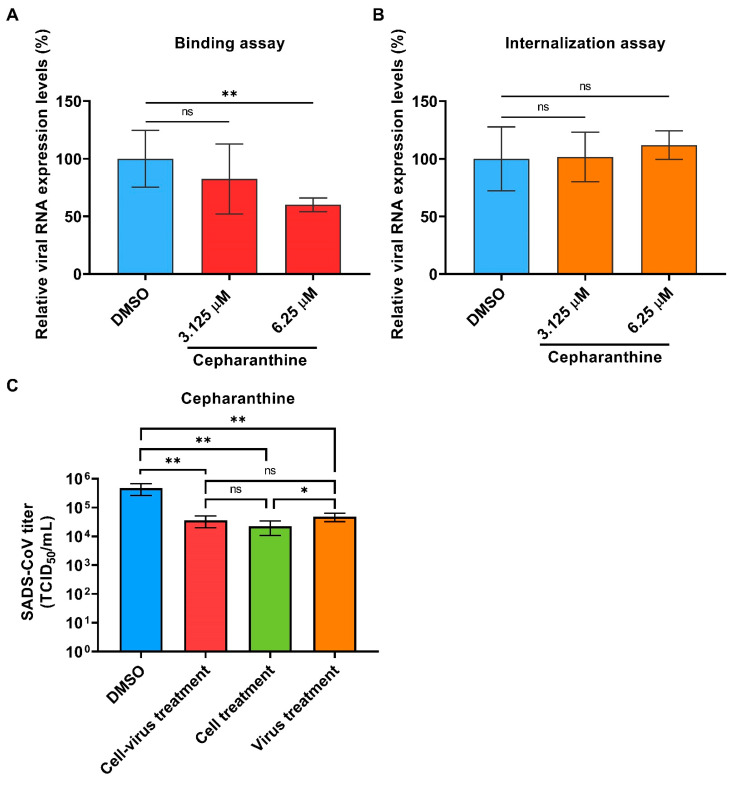
Cepharanthine inhibits SADS-CoV entry. (**A**) The effect of cepharanthine on SADS-CoV binding. Cepharanthine and SADS-CoV (MOI: 2) were incubated with precooled cells for 2 h at 4 °C. Cells were washed with cold PBS three times, and RNA was extracted for qPCR quantification. (**B**) The effect of cepharanthine on SADS-CoV internalization. Precooled cells were infected with SADS-CoV for 2 h at 4 °C and incubated with cepharanthine for 1 h at 37 °C after PBS wash. RNA was extracted for qPCR examination after proteinase K treatment. (**C**) The assessment of SADS-CoV titer at 24 hpi with different cepharanthine treatments before infection. Cells and viruses were, respectively, pre-treated with cepharanthine according to the different treatment groups, and cells were further cultured for 24 h without cepharanthine after SADS-CoV infection. The supernatant samples were harvested for TCID_50_ assay. DMSO was added as the positive control. The experiments were repeated at least twice. Error bars represent ±1 SD; ns, no significant difference; * *p* < 0.05, ** *p* < 0.01 by Student’s *t*-test.

Cepharanthine is a natural alkaloid extracted from Stephania cepharantha Hayata plant, which has been approved for a variety of acute and chronic disease treatments in Japan since the 1950s [13]. It has also been reported that cepharanthine exhibits strong antiviral activities against several viruses such as HIV, HBV, HSV1, HCoV-OC43, SARS, and SARS-CoV-2 by inhibiting viral entry and replication [14]. The results of the time-of-addition assay suggested that the anti-SADS-CoV activity of cepharanthine is mainly effective at the viral entry stage (Figure 4B). To validate the effect of cepharanthine on SADS-CoV entry, we performed binding and internalization assays with SADS-CoV in Huh7 cells. The detection of viral RNA by qPCR showed that the expressions of viral RNA could not be significantly inhibited with the cepharanthine of 3.125 μM, but reduced under the addition of 6.25 μM cepharanthine indicating the inhibition effect on virus binding (Figure 5A). However, the internalization assay demonstrated less function of cepharanthine on SADS-CoV infection (Figure 5B). We then tried to explore the exact function of cepharanthine against SADS-CoV entry with different treatments. Cepharanthine was, respectively, pre-incubated with cells, virus or both cell and virus before infection, and infected cells were continuously cultured to 24 h without compound addition, then supernatant samples were collected for virus assessment. The results showed that viral titer was decreased when cell and virus were both pre-incubated with cepharanthine before infection, and the cell pre-treated only was also effective, indicating cepharanthine may inhibit SADS-CoV entry by functioning with cells (Figure 5C). Although the virus pre-incubated treatment showed a reduction in SADS-CoV viral progeny after 24 hpi, whether cepharanthine exactly works on the virus cannot be determined, and cepharanthine remains in the virus-drug mixture were not excluded to effect cells. Moreover, we examined the expressions of virus RNA level in the supernatant suggested cepharanthine could not inhibit SADS-CoV replication when added after virus entry (Figure S2).

### 2.4. Methylene Blue Is an Inhibitor for SADS-CoV Infection

**Figure 6:**
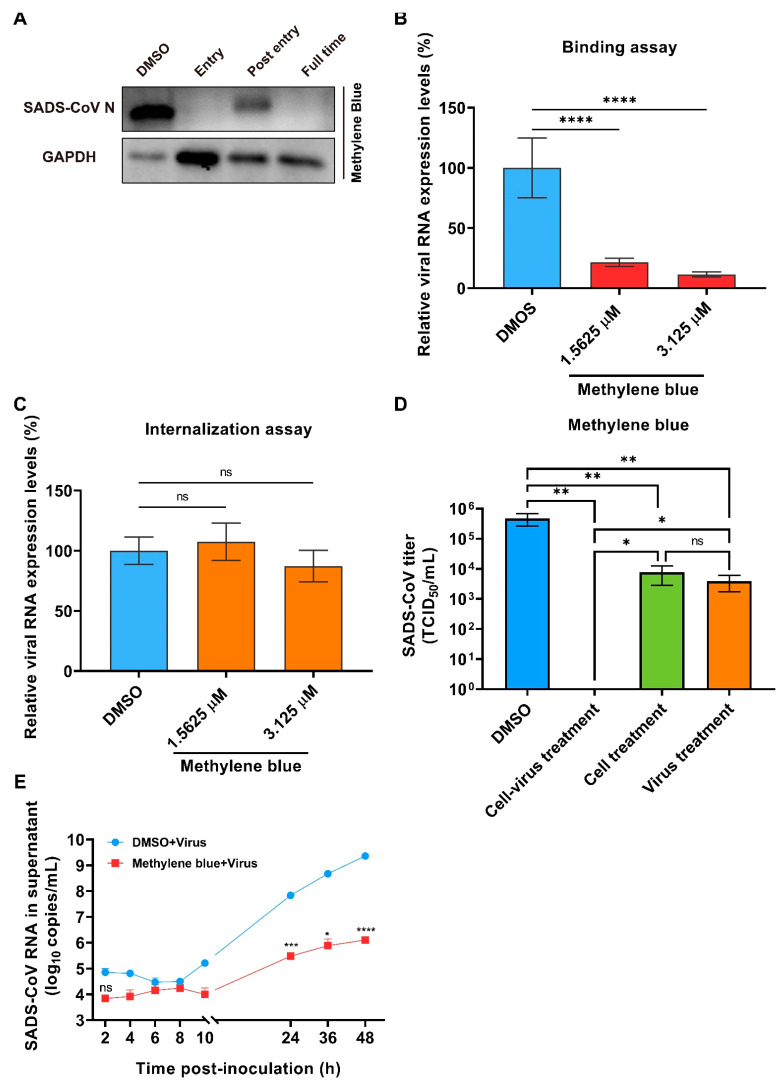
Methylene blue inhibits SADS-CoV infection. The proteins in the cells were collected from the time-of-addition assay and were tested for the expressions of viral proteins (**A**). The inhibitory effect of methylene blue on SADS-CoV binding (**B**) and internalization (**C**) were evaluated. SADS-CoV titer was tested by TCID_50_ after 24 hpi when different methylene blue treatments were used before infection (**D**). The growth kinetics of SADS-CoV in the presence of methylene blue (1.5625 μM) was also examined (**E**). DMSO was added as the positive control. The experiments were repeated at least twice. Error bars represent ±1 SD; ns, no significant difference; * *p* < 0.05, ** *p* < 0.01, *** *p* < 0.001, **** *p* < 0.0001 by Student’s *t*-test.

Methylene Blue, as a thiazine dye and an FDA-approved drug, has historically been used as an antidote, disinfectant for virus inactivation in blood products and antiviral agent for viruses, including Dengue virus (DENV), Zika virus (ZIKV), and SARS-CoV-2 [15,16]. In the time-of-addition assay, methylene blue was found to inhibit SADS-CoV entry and replication (Figure 4A), which was also validated by the detection of the viral protein expression (Figure 6A). We then performed the binding assay and found that methylene blue could significantly reduce the expression level of SADS-CoV RNA with a decrease of 78.5% and 88.5%, respectively, by the addition of methylene blue of 1.563 and 3.125 μM, indicating the inhibition of SADS-CoV binding (Figure 6B). Nevertheless, the internalization assay showed no distinct effect on viral internalization with methylene blue (Figure 6C). We applied different treatments to assess methylene blue against SADS-CoV entry (the same treatments as described for cepharanthine) and found that no matter cell or virus pre-incubation could both inhibit SADS-CoV infection, while the inhibition effect got better with cell and virus pre-incubation at the same time (Figure 6D). We also monitored SADS-CoV growth in the presence of methylene blue to investigate its effects after the viral entry. It was obviously found that methylene blue distinctly prevented SADS-CoV growth, and the inhibitory effect increased over time, with 2- to 3-log reduction of viral RNA from 24 to 48 hpi compared with the positive control (Figure 6E).

### 2.5. Effects of Gemcitabine, Mycophenolate mofetil, and Mycophenolic Acid after SADS-CoV Entry

**Figure 7:**
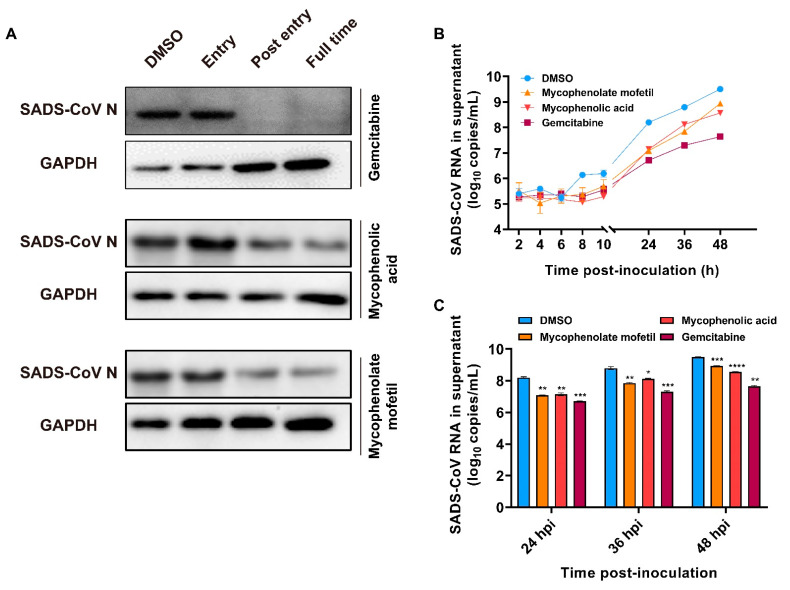
Gemcitabine, mycophenolate mofetil, and mycophenolic acid can inhibit SADS-CoV replication. (**A**) The expressions of viral proteins in cells after 48 h SADS-CoV infection with different treatments. (**B**) The growth kinetics of SADS-CoV in the presence of gemcitabine (6.25 μM), mycophenolate mofetil (12.5 μM), and mycophenolic acid (12.5 μM), respectively. (**C**) The expression of SADS-CoV RNA in the supernatant at 24 hpi, 36 hpi, and 48 hpi in the presence of indicated compounds. DMSO was added as the positive control. The experiments were repeated at least twice. Error bars represent ±1 SD, * *p* < 0.05, ** *p* < 0.01, *** *p* < 0.001, **** *p* < 0.0001 by Student’s *t*-test.

Gemcitabine is a nucleoside analogue approved by the FDA and is used as a broad-spectrum antiviral drug by interfering with viral nucleoside synthesis [17]. Regarding mycophenolate mofetil (MMF) and mycophenolic acid (MPA), MMF is a prodrug of MPA and both of them are immunosuppressants used to prevent rejection of transplant [18]. MMF and MPA have been reported to have antiviral activity against Hepatitis C Virus (HCV), human herpes virus, DENV, and SARS-CoV-2 [19,20,21,22]. We found that the addition of gemcitabine, MMF, and MPA could significantly inhibit SADS-CoV infection and replication, especially by inhibiting the post-entry stages (Figure 4C–E). To this end, we examined the SADS-CoV infection 48 h after the drugs were added and the immunoblot results showed the inhibition effect of gemcitabine, MMF and MPA on SADS-CoV replication after virus entry (Figure 7A). Compared with that of the positive control, the viral RNA in the supernatant was reduced in the presence of gemcitabine, MMF, and MPA, with 1.8-, 0.5-, and 0.9-log decrease at 48 hpi, respectively (Figure 7B,C). In addition, we also tested their effects on virus binding and internalization and showed no statistically significant difference compared with that of the positive control (Figure S3).

## 3. Discussion

With the advent of the current COVID-19 pandemic, zoonotic viruses such as coronavirus, pose high threats to the global economy and human health. The impact of SADS-CoV spread and the possibility of SADS-CoV cross-species infection implied potential risks to human health [4,8,9]. However, most of the recent studies focus on virus etiology, genetic evolution, and clinical diagnosis, with a lack of studies on infection mechanisms, as well as effective vaccines and therapeutics.

Given the costly and lengthy process of drug development, repurposing existing medications has been considered as an effective approach in response to an emergency. In this study, we screened a total of 3523 compounds obtained from three libraries comprising approved drugs to find antiviral medicines for SADS-CoV. The primary screen results showed five compounds presenting the antiviral activity to SADS-CoV, including methylene blue, cepharanthine, gemcitabine, mycophenolate mofetil, and mycophenolic acid. They exhibited inhibitory effects in a dose-dependent manner, and the selectivity indexes were more than 10, indicating their effectiveness and safety as antiviral drugs. We then examined the potential target of the drugs through time-of-addition assays. Moreover, we carried out plaque assays which found the addition of cepharanthine at the entry stage inhibited viral infection and methylene blue could reduce the number of infectious progeny virions during the whole lifecycle, while gemcitabine, mycophenolate mofetil, and mycophenolic acid inhibited viral replication at the post-entry stage.

Cepharanthine, as a Chinese medicine compound, has been identified to inhibit various viral infections, and we found its antiviral activity against SADS-CoV, especially during the virus entry stage. Further results demonstrated that cepharanthine could inhibit SADS-CoV entry and mainly block cell-virus binding. It has been previously reported that cepharanthine inhibited the entry of HCoV-OC43 and blocked the binding of SARS-CoV-2 S protein to the ACE2 receptor [23,24]. Moreover, cepharanthine was described to inhibit HIV-1 entry by stabilizing the membrane fluidity [25]. According to our results, we speculate that cepharanthine may act on host cells to block the interaction with the virus to resist SADS-CoV infection.

Methylene blue was found to exhibit effective anti-SADS-CoV activity in a dose-dependent manner and could reduce 78.5% SADS-CoV binding. It has been demonstrated that methylene blue could block the interaction between SARS-CoV-2 S protein and the ACE2 receptor, thereby preventing virus entry [26]. Methylene blue is also historically used to inactivate viruses for a long time, by intercalating nucleic acid strands, leading to strand break through guanosine oxidization [27]. In our study, we found that methylene blue presented an inhibitory effect whether the cell or the virus was pre-treated with it, as well as better inhibition for SADS-CoV entry when virus and cells were both pre-treated with the compound. Considering its broad-spectrum inhibitory potential, our results suggested methylene blue might inhibit SADS-CoV infection through both virus inactivation and interfering with virus-cell interaction before virus entry. Moreover, it was also found that methylene blue could also inhibit flavivirus infection by inhibiting the viral protease NS3 protein activity [16]. We confirmed that methylene blue could considerably inhibit SADS-CoV replication, with inhibition of viral protein expression and a 3-log viral RNA decrease at 48 hpi.

In this study, we also demonstrated the anti-SADS-CoV activity of gemcitabine. Gemcitabine has been proposed as a broad-spectrum antiviral drug against various RNA viruses by modulating nucleotide biosynthesis and stimulating innate immunity [28,29]. Our results showed gemcitabine significantly inhibited SADS-CoV with a very low EC_50_ value (0.1171 μM). Further experiments indicated that it had no effect on SADS-CoV entry but mainly acted after SADS-CoV entry, with the inhibition of viral growth over time (1.8-log reduction of viral RNA in the supernatant at 48 hpi); the findings were similar to previously reported results of gemcitabine having an inhibitory function in the replication of viruses [17].

For the immune suppressants MMF and MPA, we observed their well-inhibition effect against SADS-CoV. The EC_50_ of anti-SADS-CoV of MMF and MPA were 1.7 μM and 0.5597 μM, respectively, which were similar to the EC_50_ values against HCoV-OC43, HCoV-NL63, SARS-CoV, and MERS-CoV (Table S1) [30,31,32]. The time-of-addition and plaque assay results showed the inhibitory effect on SADS-CoV replication and viral progeny production. Notably, MMF and MPA have a similar structure, and MMF can be absorbed in the small intestine and metabolized to MPA, which may explain the minor differences in their antiviral activity [33]. Through the detection of viral protein expression and virus growth, MMF and MPA were demonstrated to inhibit SADS-CoV replication. It has been found that MPA may induce the expression of interferon-stimulated genes (ISGs) and acts synergistically with interferon to inhibit HCV replication [34].

Because of the limitation of SADS-CoV research, the mechanism of SADS-CoV infection still remains unknown; especially the fact that the specific host receptors have not been found yet hinders the analysis of antiviral mechanisms for related drugs. Further studies need to be conducted to make these clear.

In summary, this is the first report on drug screening for SADS-CoV. We have screened a total of 3523 compound-containing drug libraries and identified the antiviral activity of five compounds against SADS-CoV infection. Although the mechanisms of their antiviral action remain to be elucidated, this study provides new ideas for potential drugs in response to the outbreak of SADS-CoV.

## 4. Materials and Methods

### 4.1. Cell Line and Viruses

The human hepatocellular carcinoma cells (Huh7; ATCC, Manassas, VA, USA) were maintained in high glucose Dulbecco’s modified Eagle’s medium (DMEM; Gibco, Carlsbad, CA, USA) with 10% fetal bovine serum (FBS; PAN, Aidenbach, Germany) and 1% antibiotic/antimycotic (Gibco, Carlsbad, CA, USA) at 37 °C. Swine acute diarrhea syndrome coronavirus (SADS-CoV) strain CN/GDWT/2017 (Genbank accession no. MG557844) was isolated from a sick piglet in China and propagated in Huh7 cells and then tittered by plaque assay on Huh7 cells.

### 4.2. Compound Library

The Approved Drug Library, Antiviral Compound Library, and Anti-COVID-19 Traditional Chinese Medicine Compound Library containing a total of 3523 unique molecules were purchased from TargetMol for the antiviral drug screening. These libraries were described to screen drugs for other viruses before [10,11]. All these compounds were dissolved in DMSO (Solarbio, Beijing, China) at 10 mM. The potential antiviral drugs identified in this study were purchased from TargetMol (Boston, MA, USA).

### 4.3. Primary Screening for Antiviral Compounds

Huh7 cells were seeded in 96-well plates for 16–24 h prior to treatment. Compounds from the libraries were diluted to 1 mM in phosphate buffered saline (PBS; Cytiva, Marlborough, MA, USA) and added to a final concentration of 10 μM, together with SADS-CoV at a multiplicity of infection (MOI) of 0.1. The positive control constituted of the Huh7 cells treated with the virus and 0.1% DMSO while the negative control was treated with only 0.1% DMSO-containing media. The potential antiviral compounds from the primary screening were determined through the cytopathic effects (CPEs) and quantitative PCR (qPCR) analyses.

### 4.4. Viral RNA Extraction and Quantification

Viral RNA from the cells was extracted using Tissue/cell total RNA Extraction Kit (NOBELAB, Beijing, China) and reverse transcribed by Hifair^®^ Ⅱ 1st Strand cDNA Synthesis SuperMix for qPCR (YEASEN, Shanghai, China). The qPCR reactions were carried out using Hieff^®^ qPCR SYBR Green Master Mix (YEASEN, Shanghai, China) according to the manufacturer’s protocol. Relative expression levels of viral RNA were normalized to GAPDH and calculated with the 2^−∆∆Ct^ method. The inhibition rate was calculated using the following equation:

(1 − relative expression of viral RNA in drug treatment/relative expression of viral RNA in PC) × 100%.

The qPCR primers used were as follows:

SADS-CoV-F 5′-GTTGATTGTAAGGCTTGGCG-3′;

SADS-CoV-R 5′-AACCACACTTCCACTCAGC-3′;

GAPDH-F 5′-AGCCTCAAGATCATCAGCAATG-3′;

GAPDH-R 5′-ATGGACTGTGGTCATGAGTCCTT-3′.

### 4.5. Antiviral and Cytotoxicity Assays

The cells were seeded at a density of 1.5 × 10^4^ cells per well in 96-well plates and incubated at 37 °C overnight. To validate the antiviral potential of the screened compounds, cells were treated with a mixture of the series of two-fold diluted drugs and virus at an MOI of 0.1, while the positive control consisted of cells treated with 0.1% DMSO and virus. CPE was assessed visually at 48 hpi and the cellular RNA was collected for qPCR analysis. For cell viability assays, cells were incubated with different concentrations of the drugs for 48 h at 37 °C, and the cell viability was assessed with BioTek microplate reader (Winooski, VT, USA) using CellTiter-Blue (Promega, Fitchburg, WI, USA) according to the manufacturer’s instructions. EC_50_ and CC_50_ values were calculated by using nonlinear regression analysis.

### 4.6. Time-of-Addition Assay

According to the virus lifecycle (e.g., virus attachment, fusion, transcription, and budding) [12], Huh7 cells were treated with drugs at different stages of virus infection. For “Entry” and “Full time” treatments, cells were incubated with the drugs for 2 h at 37 °C before infection. The cells were infected with the virus at an MOI of 0.1 together with the drugs in “Entry” and “Full time” treatments, while 0.1% DMSO-virus mixture was added in the “Post entry” treatment. After 2-h infection, the supernatant from the “Entry” treatment was removed and supplemented with media containing 0.1% DMSO, and the fresh drug-containing media were used to replace the media in “Post entry” and “Full time” treatments. DMSO was added as the positive control and no virus or DMSO was treated in the Blank group. After incubation for 48 h, cellular RNA and the supernatant from all experiment groups were collected for qPCR and plaque assay, respectively.

### 4.7. Plaque Assay and TCID_50_ Assay

The Huh7 cells were seeded in 6-well plates and were then infected with ten-fold serial viral dilutions (10^−1^ to 10^−6^) and incubated for 2 h. The supernatant was discarded and 2 mL DMEM media supplemented with 2% FBS and 1% low melting-point-agar was added to each well. Cells were incubated for 3 d at 37 °C and 5% CO_2_, followed by the addition of 4% paraformaldehyde (Solarbio, Beijing, China) to fix the cells. The cells were stained with crystal violet for 5 min and then rinsed with water gently to visualize plaques. For TCID_50_ assays, Huh7 cells were seeded in 96-well plates for 16–24 h prior to experiments. The collected supernatant samples were serially ten-fold diluted and added to the cells. After 72 h incubation, the virus titer was calculated using the Muench and Reed method.

### 4.8. Western Blotting

Proteins were harvested with radioimmunoprecipitation assay lysis buffer (Beyotime, Shanghai, China) and boiled at 100 °C for 10 min together with loading buffer (TransGen Biotech, Beijing, China) after concentration determination. The proteins were separated by 10% SDS-PAGE and transferred to an Immuno-Blot polyvinylidene difluoride membrane (PVDF; Millipore, Burlington, MA, USA) using Trans-Blot SD semi-dry transfer cell (Bio-rad, Hercules, CA, USA). After being blocked with 5% skim milk, the membranes were incubated with primary antibodies at 4 °C overnight, followed by secondary antibodies for 1 h at room temperature. After being washed 3 times with TBST (20 mM Tris-HCl, 150 mM NaCl, 0.05% [*v*/*v*] Tween 20), the membrane was incubated with Immobilon Western Chemiluminescent HRP Substrate (Millipore, Burlington, MA, USA) and the signal was detected using ChemiDoc XRS^+^ imaging system (Bio-rad, Hercules, CA, USA). Primary antibodies were used as follows: SADS-CoV nucleocapsid protein was detected by SADS-CoV N protein-specific monoclonal antibody (1:1000 dilution, generously provided by Professor Jingyun Ma, College of Animal Science, South China Agricultural University, Guangzhou, China); Proteins of the housekeeping gene GAPDH was detected by anti-GAPDH mouse antibody (1:20,000 dilution, catalog number: #60004-1-1g, Proteintech, Sankt Leon-Rot, Germany). Secondary antibody was goat anti-mouse IgG HRP (1:10,000 dilution, catalog number: #SA00001-1, Proteintech, Sankt Leon-Rot, Germany). The original images for western blotting assays in the main figures were provided in Figure S4.

### 4.9. Virus Binding and Internalization Assay

Cells were precooled and infected with SADS-CoV at an MOI of 2 for 2 h at 4 °C. The supernatant was removed, and cold PBS was used to wash the cells three times. Viral RNA was extracted from the cells for qPCR quantification. For the internalization assay, infected cells as described above were further cultured for 1 h at 37 °C after PBS wash and treated with Proteinase K to remove the non-internalized virions. The cells were then lysed for RNA extraction and qPCR quantification. The indicated concentrations of compounds were separately added to the cells during the virus binding and internalization period for the antiviral efficacy assay.

### 4.10. Examination of Compounds Effect on Virus Entry

The Huh7 cells were seeded in 48-well plates before experiments. Cells were divided into four groups with different treatments: cell treatment, virus treatment, cell-virus treatment, and the positive control were treated with DMSO. For cell treatment group, only cells were incubated with compounds for 2 h at 37 °C; For virus treatment group, only viruses were incubated with compounds for 2 h at 37 °C; For cell-virus treatment group, both cells and viruses were incubated with compounds for 2 h at 37 °C, respectively. After pre-incubation, the supernatant of cell culture from all groups was discarded and washed with PBS, and then the compound pre-incubated or no-compound incubated virus dilutions were added to corresponding groups at an MOI of 2 for 2 h. Then, after the supernatant was discarded and the PBS wash was completed, fresh media were added for further incubation without any compounds. At 24 hpi, the supernatant samples were collected for TCID_50_ test.

### 4.11. Virus Growth Kinetics Assay

Pre-seeded cells were infected with SADS-CoV at an MOI of 0.1 for 2 h. After PBS wash, fresh media were added to the cells with the indicated compounds for further incubation at 37 °C. The time at which the compounds were added was marked as 0 h. The supernatant was harvested at the indicated time for qPCR quantification.

### 4.12. Statistical Analysis

In this study, Student’s *t*-test was used to determine the statistical significance between groups, and the symbols are defined as follows: * *p* < 0.05, ** *p* < 0.01, *** *p* < 0.001, **** *p* < 0.0001; ns, no significant difference.

## Figures and Tables

**Table 1:** The summary of anti-SADS-CoV activity tested in vitro.

Compound Name	CAS No.	Bioactivity	SADS-CoV		
			EC_50_ (μM)	CC_50_ (μM)	SI
Methylene blue	61-73-4	Antidote, disinfectant, antiviral	0.2175	>25	>114.94
Cepharanthine	481-49-2	Antiinflammatory, antineoplastic, antiviral	0.789	16.18	20.51
Gemcitabine	95058-81-4	Antineoplastic, antiviral	0.1171	88.48	755.59
Mycophenolate mofetil	115007-34-6	Immune suppressant, antineoplastic, antiviral	1.7	>100	58.82
Mycophenolic acid	24280-93-1	Immune suppressant, antineoplastic, antiviral	0.5597	>100	178.67

## Data Availability

Not applicable.

## Supplementary Materials

The following supporting information can be downloaded at: https://www.mdpi.com/article/10.3390/ijms231911250/s1.
