# Supplementary material for: Antiviral Drugs Screening for Swine Acute Diarrhea Syndrome Coronavirus
Source: Int J Mol Sci. 2022 Sep 24;23(19):11250. doi: 10.3390/ijms231911250 (PMC9569988; doi:10.3390/ijms231911250)
Supplement: Supplementary file 1 [file ijms-23-11250-s001.zip › ijms-1884986-supplementary.pdf]

Figure S1 Cytopathic effects of SADS-CoV infection in the presence of the five drugs.

Methylene blue (1.5625 μM), mycophenolate mofetil (12.5 μM), mycophenolic acid (12.5 μM), gemcitabine (6.25 μM) and cepharanthine (6.25 μM) were separately added at different stages of SADS-CoV infection. The representative images of CPE on Huh7 cells after 48 hpi were shown.

Figure S2 The effect of cepharanthine on SADS-CoV growth after virus entry.

Cepharanthine (6.25 μM) was added after SADS-CoV entry and the supernatant samples were collected to assess the virus growth by qPCR.

Figure S3 The effects of gemcitabine, mycophenolate mofetil and mycophenolic acid on SADS-CoV binding and internalization.

Gemcitabine, mycophenolate mofetil and mycophenolic acid were tested the effects on SADS-CoV binding (A−C) and internalization (D−F). The experiments were repeated at least twice. Error bars represent ±1 SD; ns, not significant.

Figure S4 Original images for western blotting assays in main figures.

Table S1 Antiviral activity of mycophenolate mofetil and mycophenolic acid.

Previous studies were described about mycophenolate mofetil and mycophenolic acid against other viruses. -: Not test

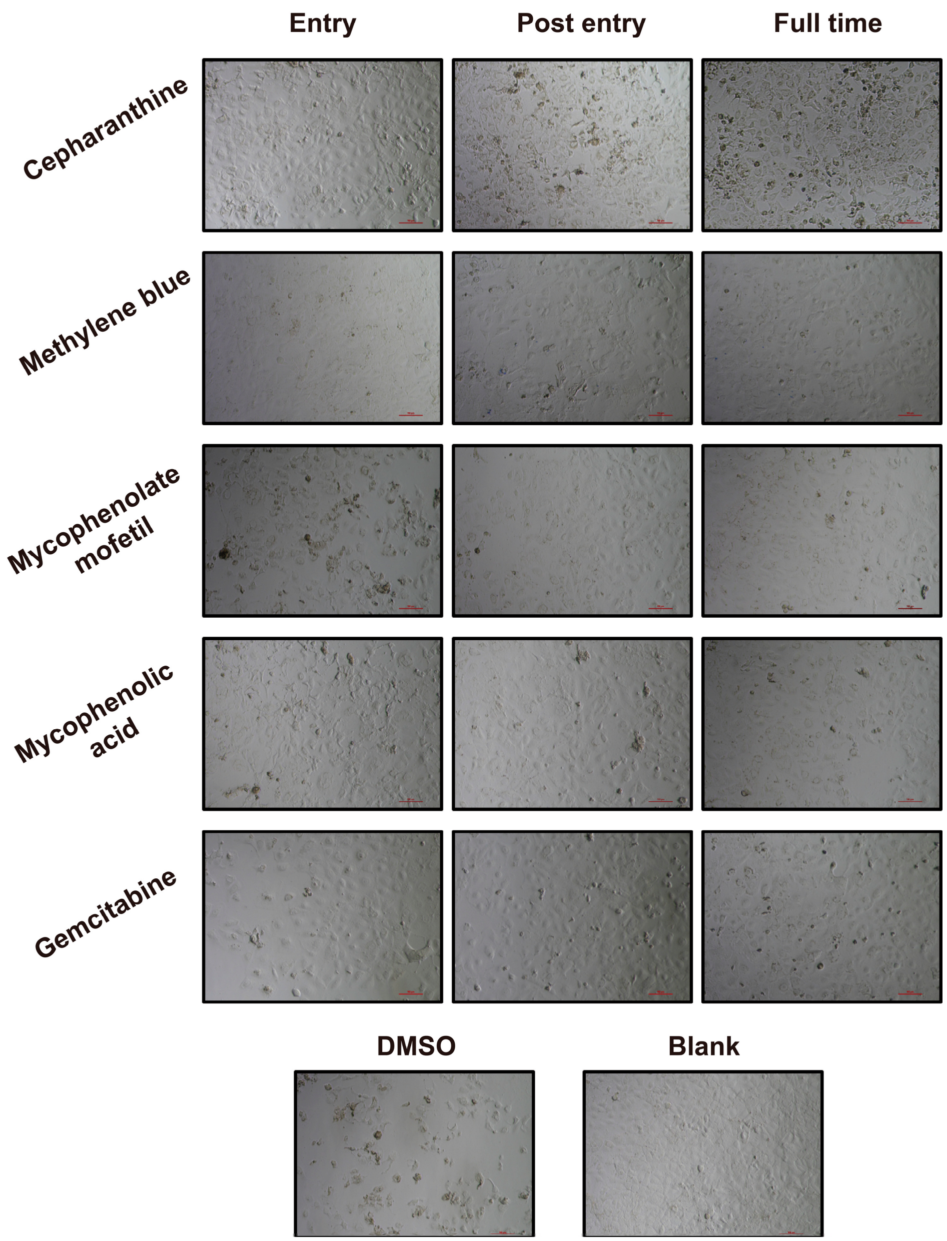

Figure S1. Cytopathic effects of SADS-CoV infection in the presence of the five drugs.

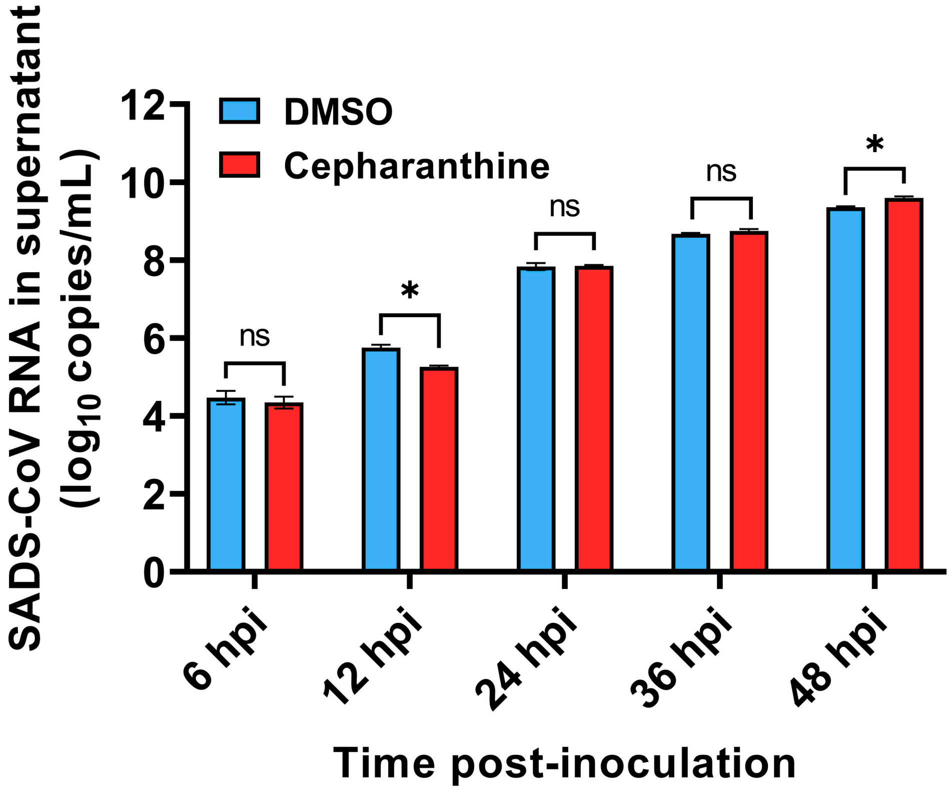

Figure S2. The effect of cepharanthine on SADS-CoV growth after virus entry.

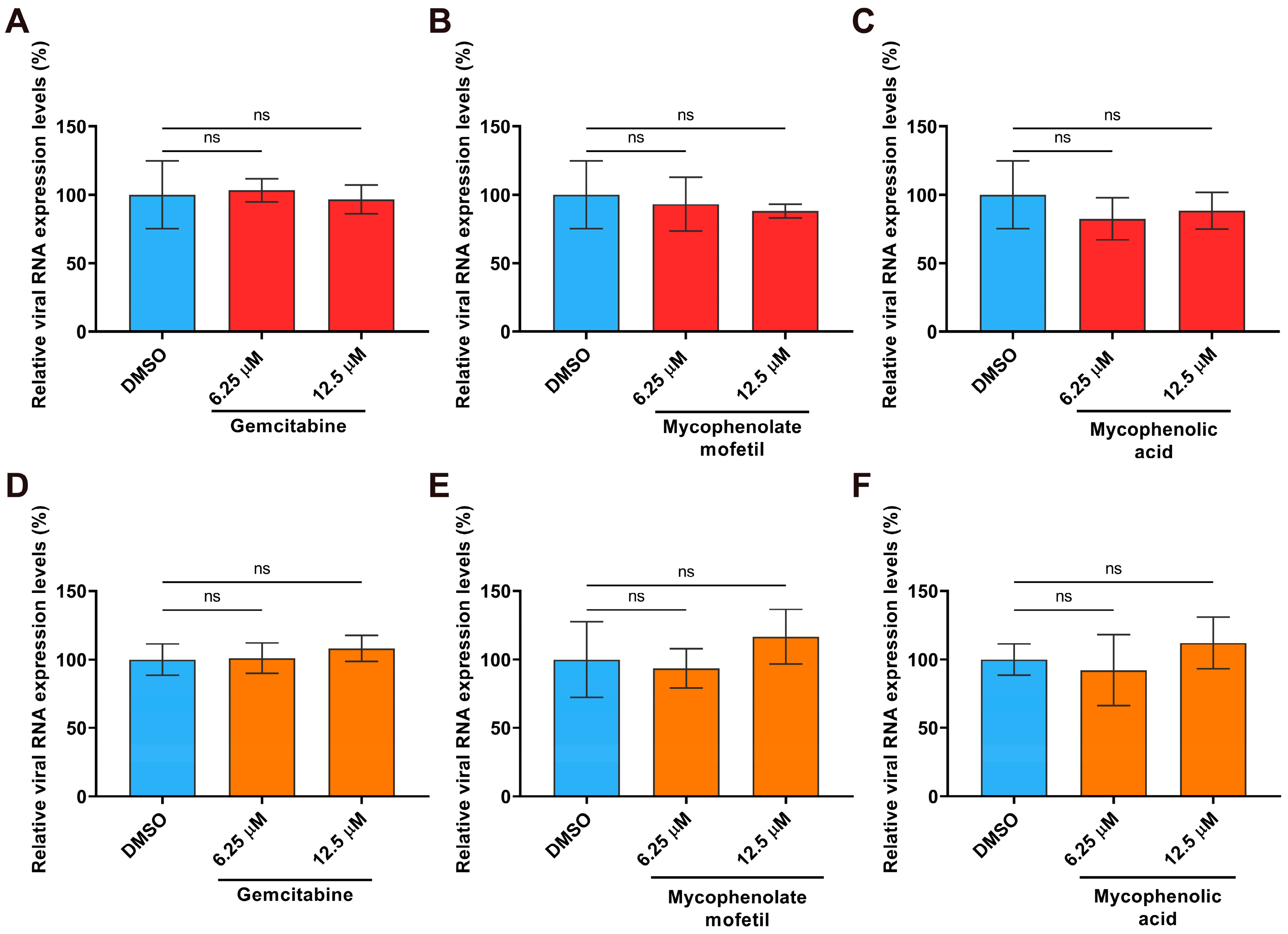

Figure S3. The effects of gemcitabine, mycophenolate mofetil and mycophenolic acid on SADS-CoV binding and internalization.

Figure 6A

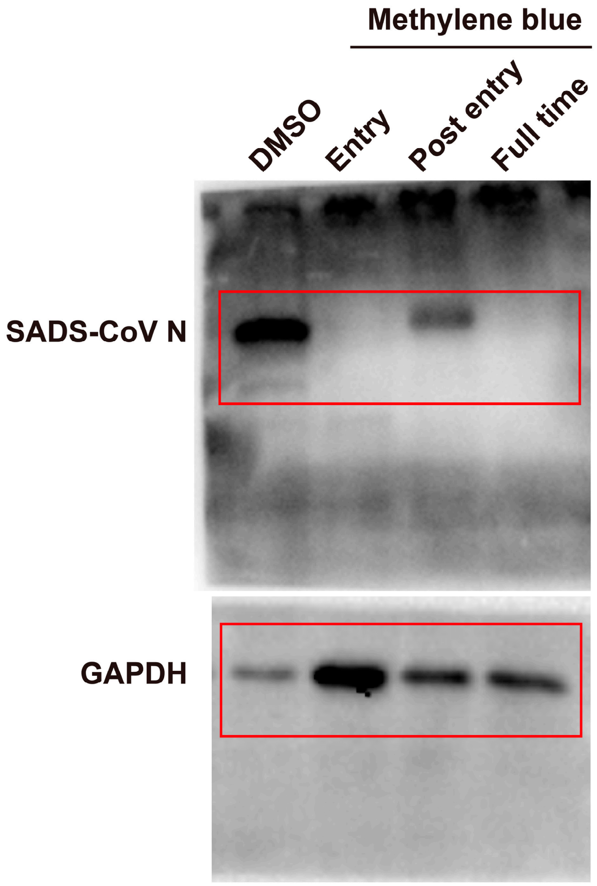

Figure 7A

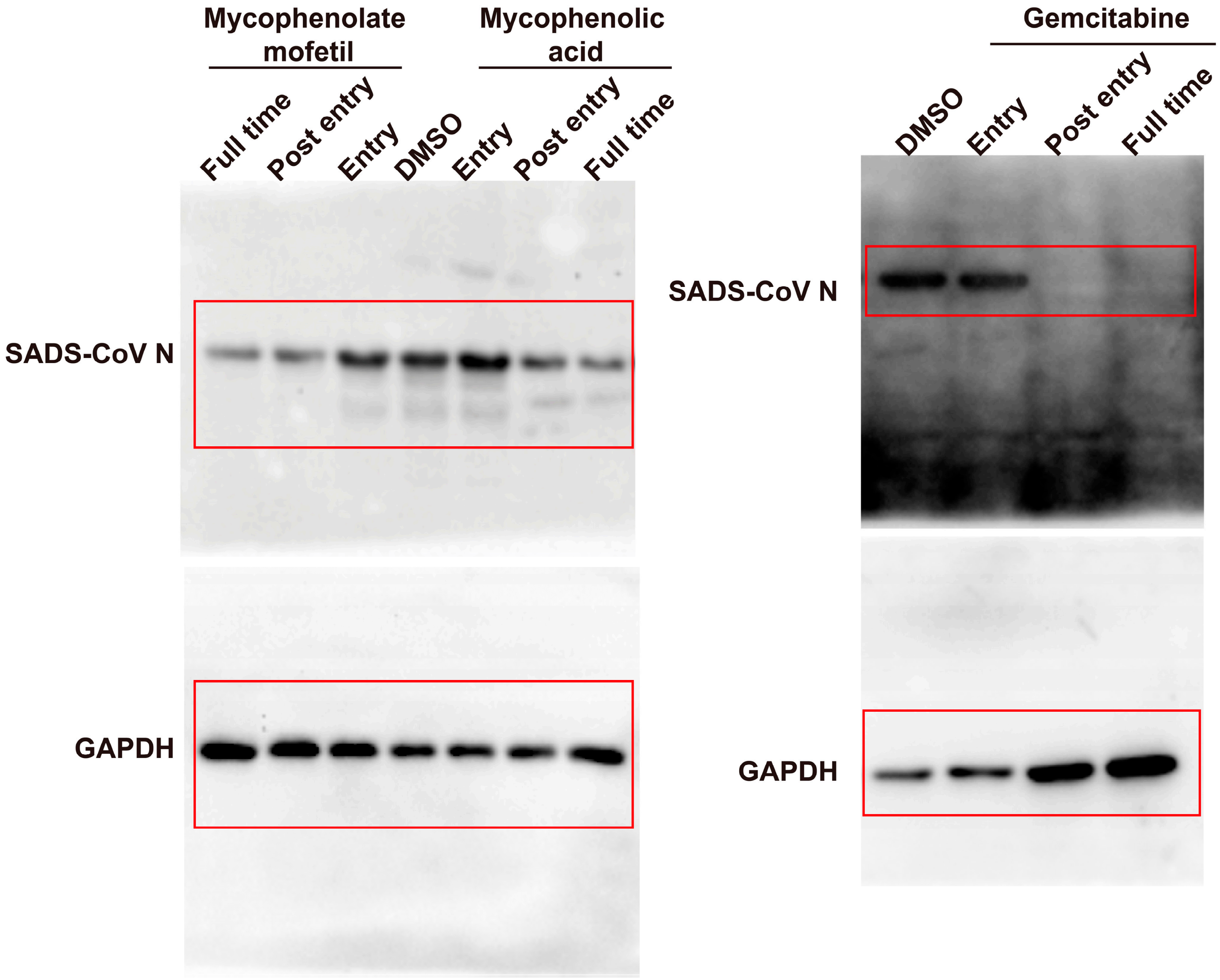

Figure S4. Original images for western blotting assays in main figures.

Table S1. Antiviral activity of mycophenolate mofetil and mycophenolic acid against several viruses.

-: Not test

Virus	Mycophenolate mofetil	Mycophenolic acid
	EC_{50} (μM) / CC_{50} (μM)	EC_{50} (μM) / CC_{50} (μM)
**HCoV-OC43** [30]	**1.58 / 3.43**	**1.95 / 3.55**
**HCoV-NL63** [30]	**0.23 / 3.01**	**0.18 / 3.44**
**MERS-CoV** [30]	**1.54 / 3.17**	**1.95 / 3.21**
**SARS-CoV** [31]	**-**	**>30 / >30**
**SARS-CoV-2** [32]	**-**	**0.87 / -**

References

30. Shen, L.; Niu, J.; Wang, C.; Huang, B.; Wang, W.; Zhu, N.; Deng, Y.; Wang, H.; Ye, F.; Cen, S.; Tan, W. High-Throughput Screening and Identification of Potent Broad-Spectrum Inhibitors of Coronaviruses. *J. Virol.* **2019**, *93*.
31. Barnard, D.L.; Day, C.W.; Bailey, K.; Heiner, M.; Montgomery, R.; Lauridsen, L.; Winslow, S.; Hoopes, J.; Li, J.K.; Lee, J.; Carson, D.A.; Cottam, H.B.; Sidwell, R.W. Enhancement of the infectivity of SARS-CoV in BALB/c mice by IMP dehydrogenase inhibitors, including ribavirin. *Antiviral Res.* **2006**, *71*, 53-63.
32. Kato, F.; Matsuyama, S.; Kawase, M.; Hishiki, T.; Katoh, H.; Takeda, M. Antiviral activities of mycophenolic acid and IMD-0354 against SARS-CoV-2. *Microbiol. Immunol.* **2020**, *64*, 635-639.
